# Extracellular Vesicles Derived from Human CD24^+^ Dental Papilla Stem Cells Promote Vascularized Dental Pulp Regeneration

**DOI:** 10.3390/biom16030390

**Published:** 2026-03-05

**Authors:** Jie Li, Tian Chen, Cheng Liang, Peini Lin, Weidong Tian, Zhi Liu, Lei Liu

**Affiliations:** 1 State Key Laboratory of Oral Diseases, National Clinical Research Center for Oral Diseases, Engineering Research Center of Oral Translational Medicine, Ministry of Education, National Engineering Laboratory for Oral Regenerative Medicine, West China Hospital of Stomatology, Sichuan University, Chengdu 610041, China; lijie@stu.scu.edu.cn (J.L.); tchen0629@scu.edu.cn (T.C.); liangcheng@stu.scu.edu.cn (C.L.); linpeini@stu.scu.edu.cn (P.L.); drtwd@scu.edu.cn (W.T.); 2 Department of Oral & Maxillofacial Surgery, West China Hospital of Stomatology, Sichuan University, Chengdu 610041, China

**Keywords:** pulp regeneration, extracellular vesicles, CD24^+^ hDPCs, angiogenesis

## Abstract

Pulp necrosis remains a significant clinical challenge in dentistry, as current therapeutic approaches fail to achieve functional pulp regeneration. Extracellular vesicles (EVs), as crucial mediators of intercellular communication, offer new opportunities for regenerative strategies. In this study, we focus on CD24^+^ human dental papilla cells (CD24^+^ hDPCs), a functionally defined subpopulation previously characterized as having superior regenerative potential, and evaluate the regenerative potential of their derived EVs (CD24^+^ EVs) in pulp-like tissue regeneration. CD24^+^ EVs significantly enhanced the proliferation, migration, and osteo/odontogenic differentiation of human dental pulp stem cells (hDPSCs) and markedly promoted endothelial tube formation in vitro. In a treated dentin matrix (TDM)-based ectopic regeneration model, CD24^+^ EVs increased cellular accumulation within the regenerated tissue and robust angiogenesis, inducing the formation of well-organized, highly vascularized pulp-like tissue with dense cellular architecture and positive DSPP expression. Together, these findings suggest that CD24^+^ EVs concurrently enhance cell migration, odontogenic differentiation, and angiogenesis, and support a promising cell-assisted EV strategy grounded in functionally defined cellular subpopulations for pulp-like tissue regeneration.

## 1. Introduction

Dental caries and pulp diseases are among the most prevalent oral conditions globally and significantly impair quality of life [1]. At present, root canal therapy remains the primary clinical treatment for irreversible pulpitis and pulp necrosis, involving the removal of infected pulp and filling the canal with bioinert materials. Although this technique effectively controls infection and alleviates symptoms, it does so at the expense of pulp vitality. The treated teeth lose essential physiological functions such as biological perception, defensive repair, and continuous nutritional supply, rendering them more susceptible to structural weakening, fracture, and eventual tooth loss, and thereby severely compromising long-term oral health [2,3]. Consequently, overcoming current therapeutic limitations and developing alternative approaches capable of reconstructing pulp structure while restoring biological functions has emerged as a critical challenge in regenerative endodontics.

With advances in tissue engineering and regenerative medicine, stem cell-based strategies utilizing odontogenic stem cells, particularly dental pulp stem cells (DPSCs) and stem cells from the apical papilla (SCAPs), have emerged as promising approaches for pulp regeneration [4,5]. These cells exhibit outstanding self-renewal capacity and intrinsic osteo/odontogenic differentiation potential, positioning them as strong candidates for pulp tissue reconstruction [6]. Despite their potential, stem cell therapies face significant hurdles in clinical translation due to safety concerns regarding cell transplantation and the complex logistical requirements of viable cell management [7,8,9]. Consequently, these practical constraints have driven increasing interest in safer and more controllable strategies. Notably, the persistence of functional stem cells within the apical region or residual vital pulp tissue following pulp extirpation provides a biological basis for developing cell-assisted EV approaches aimed at pulp regeneration [10,11,12].

In recent years, extracellular vesicles (EVs) have emerged as important mediators of intercellular communication, carrying diverse bioactive molecules such as proteins, microRNAs, and glycoconjugates that reflect the characteristics of their cells of origin and participate in the regulation of tissue regeneration [13,14,15]. Compared with stem cell transplantation, EV-based approaches can recapitulate part of the beneficial biological effects of parental cells while offering low immunogenicity, easier storage, and a superior safety profile, making them promising strategies. However, accumulating evidence has revealed pronounced heterogeneity within conventionally stem cell populations [16,17,18]. Distinct subpopulations display substantial differences in self-renewal capacity, differentiation potential, and paracrine activity. These variations result in heterogeneous EV cargo profiles, which may contribute to the variable efficacy and poorly understood mechanisms observed in studies utilizing mixed stem cell sources. Therefore, identifying and harnessing specific subpopulations with superior functional properties may help to address these limitations and improve the transplantation of dental pulp regeneration therapies.

In our previous work, we identified a CD24a dental mesenchymal subpopulation in mice that acts as a progenitor population for pulp dentin complex formation with enhanced regenerative potential [19,20]. However, the biological activities of EVs derived from this specific subpopulation and their capacity to induce pulp regeneration have remained unexplored. In this study, we performed integrated in vitro and in vivo experiments, using co-culture systems, conditioned medium (CM) treatment, and a treated dentin matrix (TDM)-based ectopic regeneration model to systematically evaluate the biological functions and regenerative effects of CD24^+^ hDPCs-derived extracellular vesicles (CD24^+^ EVs) [21,22]. Our findings showed that CD24^+^ EVs significantly enhanced the proliferation, migration, and osteo/odontogenic differentiation of human dental pulp stem cells (hDPSCs), while also effectively promoting endothelial cell migration and tube formation. In vivo, CD24^+^ EVs promoted cell migration and supported the formation of well-structured, highly vascularized pulp-like tissue with characteristic odontogenic differentiation markers, suggesting that CD24^+^ EVs preserve the superior regenerative properties as crucial acellular agents for efficient pulp regeneration. Collectively, this study highlights, at the subpopulation level, the therapeutic potential of CD24^+^ EVs in pulp regeneration and supports a promising and cell-assisted EV strategy for pulp regeneration.

## 2. Materials and Methods

### 2.1. Cell Culture

All procedures were strictly performed in accordance with the protocols and guidelines approved by the Medical Ethics Committee of West China Hospital of Stomatology, Sichuan University (Approval No. WCHSIRB-CT-2025-318). Human dental papilla tissues were obtained from extracted third molars of donors (aged 13–25 years) who provided informed consent. The teeth were collected for orthodontic treatment or surgical purposes. The tissues were sequentially immersed in phosphate-buffered saline (PBS, Cyagen Biosciences, Suzhou, China) containing 10%, 5%, and 2% penicillin/streptomycin (P/S, Gibco, Grand Island, NY, USA), each for 3 min. The tissues were then minced into approximately 1 mm^3^ fragments using ophthalmic scissors and digested with 3 mg/mL collagenase type I (Sigma-Aldrich, St. Louis, MO, USA) at 37 °C for 30 min. The digested tissue fragments were transferred to 25 cm^2^ culture flasks (Corning Inc., Corning, NY, USA) containing α-MEM basal medium (Gibco) supplemented with 10% fetal bovine serum (FBS; Gibco) and 1% P/S. The flasks were incubated at 37 °C in a 5% CO_2_ incubator for explant culture. The medium was refreshed every three days. When cells reached 80–90% confluence, the cells were passaged using trypsin (Gibco).

Human dental pulp tissues were collected from extracted third molars of donors (aged 18–25 years). The subsequent procedures were identical to those described for hDPCs isolation. The human umbilical vascular endothelial cells (HUVECs) were purchased from ATCC (Manassas, VA, USA) and cultured in F12/DMEM medium (Gibco) supplemented with 10% FBS and 1% P/S. The cells were maintained at 37 °C in a 5% CO_2_ incubator. Other culture procedures were similar to those used for hDPCs and hDPSCs. All subsequent experiments were conducted using P2–P6 cells.

### 2.2. Phenotypic Characterization and Cell Sorting

#### 2.2.1. Flow Cytometric Analysis

P3 hDPCs in good growth condition were harvested to prepare single-cell suspensions (>2 × 10^5^ cells per group). The cells were stained with the following fluorescently labeled antibodies in PBS containing 2% FBS at 4 °C for 30 min protected from light: CD90-FITC (BD, Franklin Lakes, NJ, USA), CD29-PE (BD), CD44-FITC (BD), CD34-PE (BD), and CD45-FITC (BD). Isotype-matched antibodies served as negative controls. After washing with PBS, samples were analyzed using an Accuri C6 flow cytometer (BD), and data were processed with FlowJo V10 software (BD).

#### 2.2.2. Sorting of CD24^+^ and CD24^−^ hDPCs

P2-P3 hDPCs in good growth condition were harvested and prepared as single-cell suspensions. The cells were labeled with CD24-FITC antibody (BD) and incubated at 4 °C for 30 min, protected from light. After washing with PBS, CD24^+^ and CD24^−^ hDPCs were isolated using a FACS Aria II cell sorter (BD) into separate collection tubes. Data analysis was performed using FlowJo V10 software.

### 2.3. Preparation of CM

After reaching approximately 80% confluence, the cells were gently washed twice with PBS and then maintained in serum-free α-MEM basal medium. After 48 h, the culture supernatant was collected and centrifuged at 800× *g* for 10 min to remove detached cells. The supernatant was then filtered through a 0.22 μm membrane for sterilization. The filtrate, designated as CM, was aliquoted and stored at −80 °C for subsequent experiments. The CM derived from CD24^+^ and CD24^−^ hDPCs were labeled as CD24^+^ CM and CD24^−^ CM, respectively.

### 2.4. Cell Functional Assays

#### 2.4.1. Cell Proliferation Assay

- CCK-8 Assay: The cells were seeded in 96-well plates (Corning) at a density of 1 × 10^4^ cells per well. After 1, 3, and 5 days of culture, 10 μL of CCK-8 solution was added to each well according to the manufacturer’s instructions. Following 1 h of incubation, the absorbance at 450 nm was measured using a microplate reader.
- EdU Cell Proliferation Assay: hDPSCs in the logarithmic growth phase were resuspended in α-MEM containing 20 μg/mL of CD24^+^ EVs or CD24^−^ EVs and seeded into confocal dishes at 2 × 10^5^ cells per well. After 24 h of culture, EdU (Beyotime Biotechnology, Shanghai, China) was added and incubated at 37 °C for 2 h according to the manufacturer’s instructions. The cells were then fixed with 4% paraformaldehyde (Sigma-Aldrich) and permeabilized with 0.5% Triton X-100 (Sigma-Aldrich). EdU detection was performed using the click reaction solution (containing Alexa Fluor 488-azide) provided in the kit under light-protected conditions. Cell nuclei were counterstained with DAPI (Solarbio Life Sciences, Beijing, China). Images were acquired using a fluorescence microscope (Olympus, Tokyo, Japan), and the percentage of EdU-positive cells was quantified using ImageJ software (version 1.52a).

#### 2.4.2. Cell Co-Culture Assay

A Transwell system with 0.4 μm pore size (Corning) and 24-well plates (Corning) were used. hDPSCs (4 × 10^4^ per well) were seeded in the 24-well plate, while CD24^+^/^−^ hDPCs were seeded in the upper inserts. After an overnight culture for cell attachment, the upper inserts containing CD24^+^/^−^ hDPCs were transferred to the 24-well containing hDPSCs. The culture medium was then replaced with the required experimental medium for subsequent assays.

#### 2.4.3. Cell Migration Assessment

- Wound Healing Assay: the cells were seeded in 12-well (4 × 10^5^ per well) plates and cultured until reaching 90% confluence. A sterile 200 μL pipette tip was used to create a scratch, and detached cells were removed by washing with PBS. The medium was replaced with the experimental medium (basal medium/CM/EVs-containing medium). Images were captured at 0 and 24 h under a microscope (Olympus), and the Wound healing rate was calculated using ImageJ software.
- Transwell Migration Assay: the cells were resuspended in serum-free medium and seeded into the upper chambers of 24-well Transwell plates (Corning) at 2 × 10^4^ cells per well. The lower chambers were filled with basal medium containing 5% exosome-depleted fetal bovine serum (FBS-NE, Cyagen Biosciences, Suzhou, China). After 24 h of incubation, the inserts were removed, fixed with 4% paraformaldehyde (Sigma-Aldrich), and stained with 0.1% crystal violet (Solarbio Life Sciences). Non-migrated cells on the upper surface were gently removed with a cotton swab, and migrated cells on the lower membrane surface were observed and counted under a microscope (Olympus).

#### 2.4.4. Osteo/Odontogenic Differentiation Induction and Staining

When cells reached 60–70% confluence, the medium was replaced with osteogenic induction medium (containing 10% FBS, 50 μg/mL ascorbic acid, 10 mM β-glycerophosphate, and 100 nM dexamethasone in basal medium). For EV experiments, the FBS was replaced with FBS-NE. The medium was refreshed every 3 days.

- Alkaline phosphatase (ALP) Staining: After 7 days of induction, ALP staining was performed according to the manufacturer’s instructions (Cyagen Biosciences). The stained areas were analyzed using ImageJ software to measure optical density values for semi-quantitative assessment of ALP activity.
- Alizarin Red S (ARS) Staining: After 21 days of induction, the cells were fixed with 4% paraformaldehyde for 10 min and incubated with 40 mM ARS solution (Cyagen Biosciences) at room temperature for 30 min to detect mineralized nodule formation. For semi-quantitative analysis, 10% (*w*/*v*) cetylpyridinium chloride (Sigma-Aldrich) was added and incubated for 1 h to dissolve the bound dye, followed by measurement of absorbance at 562 nm using a microplate reader.

#### 2.4.5. HUVECs Tube Formation Assay

HUVECs were resuspended in CM or 2% FBS-NE medium and seeded into 24-well plates pre-coated with Matrigel at a density of 8 × 10^4^ cells per well. After 6 h of incubation at 37 °C, tube-like structures were observed under an inverted microscope. The number of nodes was analyzed using ImageJ software.

#### 2.4.6. RT-qPCR

Total RNA was extracted from cells using Trizol reagent (Invitrogen, Carlsbad, CA, USA), and its concentration and purity were measured. RNA was reverse-transcribed into cDNA according to the reverse transcription kit instructions (Vazyme, Nanjing, China). Using cDNA as a template, amplification was performed on a RT-qPCR system with SYBR Green (Vazyme) premix. *GAPDH* was used as the internal reference gene, and the relative expression of target genes (e.g., *CD24*, *OCN*, *OPN*) was calculated using the 2^−ΔΔCt^ method (Table S1).

### 2.5. Preparation and Characterization of EVs

When CD24^+^ hDPCs and CD24^−^ hDPCs reached 60–80% confluence, the medium was replaced with α-MEM containing 5% FBS-NE. After 48 h of culture, the supernatant was collected and sequentially centrifuged at 300× *g* for 10 min (to remove live cells), 2000× *g* for 10 min (to remove dead cells), and 10,000× *g* for 30 min (to remove cell debris). The resulting supernatant was then subjected to ultracentrifugation at 100,000× *g* for 70 min to obtain a crude EV pellet. The pellet was resuspended in PBS and further purified by another round of ultracentrifugation at 100,000× *g* for 70 min. The final EV pellet was resuspended in an appropriate volume of PBS and stored at −80 °C. The protein concentration of EVs was quantified using a BCA protein assay kit (TIANGEN Biotech, Beijing, China). The particle-to-protein ratios of CD24^+^/^−^ EVs were further assessed by nanoparticle tracking analysis (NTA) and were comparable between groups (approximately 6.08 × 10^8^ particles per µg protein).

- Transmission Electron Microscopy (TEM): Approximately 5 μL of each sample was applied to Formvar-carbon-coated electron microscopy grids and incubated at room temperature for 10 min to facilitate nonspecific particle adhesion. The grids were then washed with Milli-Q water and stained with uranyl acetate solution (pH 7.0) for approximately 3 min. Excess liquid was removed using filter paper, and the grids were air-dried. Imaging was performed using a Jeol 1010 electron microscope (JEM-1400PLUS, Tokyo, Japan).
- NTA: Samples were diluted in ultrapure water at ratios ranging from 1:100 to 20,000. All samples were measured in duplicate under consistent instrument settings. Measurements were conducted in 488 nm laser scattering mode, and data were analyzed using ZetaView software (version 8.02.31).
- Western Blot Analysis: Exosomal proteins were extracted using RIPA lysis buffer (Beyotime Biotechnology) and quantified with a BCA kit. Equal amounts of protein samples were separated by SDS-PAGE and transferred onto PVDF membranes. The membranes were blocked with 5% skim milk at room temperature for 1 h, followed by incubation with specific primary antibodies at 4 °C overnight. Subsequently, the membranes were incubated with horseradish peroxidase-conjugated secondary antibodies (Solarbio Life Sciences) at room temperature for 1 h. Finally, signals were developed using an ECL chemiluminescence substrate and captured by a chemiluminescence imaging system. The expression of exosomal marker proteins: CD63 (ZEN-BIO, Hangzhou, China), CD9 (ZEN-BIO, Hangzhou, China), TSG101 (Abcam, Cambridge, UK), HSP70 (ZEN-BIO), and the negative marker GM130 (Abcam) was detected.

### 2.6. Subcutaneous Ectopic Transplantation in Nude Mice

All experimental protocols and procedures were approved by the Ethics Committees of West China School of Stomatology, Sichuan University (Approval No. WCHSIRB-AT-2025-502).

#### 2.6.1. Preparation of Treated Dentin Matrix (TDM)

Porcine mandibles were collected from a slaughterhouse, and mandibular incisors were extracted for the preparation of TDM. Briefly, after extraction, the teeth were processed using a dental handpiece to create cylindrical hollow structures measuring 6–8 mm in length, with a wall thickness of approximately 1 mm and an inner diameter of about 4 mm. The TDM was then thoroughly rinsed in a gradient of ethylenediaminetetraacetic acid (EDTA, Cyagen Biosciences) solutions, and then the TDM was immersed in PBS containing P/S. One end of the TDM was sealed with a gutta-percha point to simulate in vivo conditions, and the samples were stored at 4 °C for later use.

- Control group: The middle part of the TDM lumen, approximately 1 mm from the opening, was filled with thermosensitive type I collagen. After incubation at 37 °C for 30 min to allow collagen solidification, the remaining lumen was filled with type I collagen containing hDPSCs resuspended at a density of 1 × 10^7^ cells/mL to mimic the physiological presence of resident stem cells in the periapical region during pulp repair. The construct was incubated at 37 °C for another 30 min to solidify.
- CD24^−^ EVs group: CD24^−^ EVs were mixed with thermosensitive type I collagen at a concentration of 50 μg/mL and filled into the middle part of the TDM lumen, approximately 1 mm from the opening. After solidification at 37 °C for 30 min, the remaining lumen was filled with type I collagen containing hDPSCs (1 × 10^7^ cells/mL) and incubated at 37 °C for 30 min to solidify.
- CD24^+^ EVs group: CD24^+^ EVs were prepared and applied following the same procedure as the control group.

#### 2.6.2. Surgical Implantation and Sample Collection

Five-week-old male BALB/c-nude mice were purchased from GemPharmatech Co., Ltd. (Nanjing, China). Mice were randomly allocated to each group, with a sample size of five animals per group (*n* = 5). Mice were anesthetized via isoflurane inhalation. The dorsal skin was disinfected, and a longitudinal incision of approximately 1 cm was made. A subcutaneous pocket was created by blunt dissection, into which the TDM composite graft was implanted. The incision was then sutured. Four weeks post-transplantation, the mice were euthanized by anesthetic overdose. The subcutaneous grafts were carefully harvested, fixed in 4% paraformaldehyde for 24 h, decalcified with EDTA, dehydrated through a graded ethanol series, and embedded in paraffin for subsequent histological analysis.

### 2.7. Immunofluorescence and Immunohistochemistry

#### 2.7.1. Cell Immunofluorescence

The cells were seeded in confocal dishes. After overnight culture, the cells were washed three times with PBS and fixed with 4% paraformaldehyde for 10–15 min at room temperature. Permeabilization was performed using PBS containing 0.3% Triton X-100 for 10 min (this step was omitted for cell surface antigens). After blocking with 5% bovine serum albumin (BSA) in PBS for 1 h, the cells were incubated with primary antibodies at 4 °C overnight. The following day, the cells were washed with PBS and incubated with corresponding fluorescently-labeled secondary antibodies (Life Technologies, Carlsbad, CA, USA) for 1 h at room temperature protected from light. Cell nuclei were counterstained with 1 mg/mL DAPI solution (Solarbio Life Sciences), and anti-fade mounting medium (Solarbio Life Sciences) was applied before imaging. Images were captured using a fluorescence confocal microscope (Olympus). In this study, immunofluorescence staining was performed to detect the expression of Vimentin (VIM, Santa Cruz Biotechnology, Dallas, TX, USA) and Cytokeratin 14 (CK14; Millipore, Burlington, MA, USA).

#### 2.7.2. Paraffin Section Preparation and Staining

Samples retrieved from in vivo experiments were fixed in 4% paraformaldehyde and then decalcified in 17% EDTA solution (pH 7.0) at 37 °C for two weeks. After dehydration through a graded ethanol series, samples were embedded in paraffin and sectioned into 5 μm-thick slices.

- H&E and Masson Staining: Performed according to the standard protocols of the respective kits (BioSS, Beijing, China) to observe tissue morphology and collagen fibers.
- Immunohistochemistry (IHC): After deparaffinization, rehydration, and antigen retrieval, sections were processed using a DAB detection kit (ZSGB-BIO, Beijing, China) according to the manufacturer’s instructions. Endogenous peroxidase activity was blocked with 3% H_2_O_2_, and non-specific binding was blocked with 5% BSA. Sections were then incubated with primary antibodies at 4 °C overnight, followed by incubation with HRP-conjugated secondary antibodies at room temperature. DAB was used for color development, and hematoxylin was used for counterstaining. Sections were mounted with neutral balsam. In this experiment, immunohistochemical staining was performed using antibodies against α-smooth muscle actin (α-SMA; Abcam) and the human-specific marker NM95 (Abcam).
- Immunofluorescence: The procedure was similar to IHC up to primary antibody incubation. Subsequently, sections were incubated with fluorescent secondary antibodies, counterstained with DAPI, and mounted with anti-fade mounting medium. In this study, immunofluorescence staining was conducted using antibodies against CD31 (Santa Cruz Biotechnology), Ki67 (Abcam), and dentin sialophosphoprotein (DSPP; Santa Cruz Biotechnology).

### 2.8. Statistical Analysis

All data were presented as the mean ± SD (≥3 biological replicates). All experiments were independently performed at least three times. For each experiment, *n* refers to independent biological samples rather than technical repeats. Statistical analyses were performed using GraphPad Prism software (version 10.42). Comparisons between two groups were analyzed using Student’s *t*-test, while statistical comparisons among multiple groups were performed using one-way ANOVA followed by Tukey’s post hoc test. These parametric tests were applied as standard approaches for comparisons of independent biological replicates in experimental studies. A value of *p* < 0.05 was considered statistically significant. Significance levels are indicated as * *p* < 0.05, ** *p* < 0.01, *** *p* < 0.001, and **** *p* < 0.0001, while *n.s*. denotes not significant.

## 3. Results

### 3.1. CD24^+^ hDPCs Exhibit Superior Mineralization Capacity Compared to CD24^−^ hDPCs

**Figure 1:**
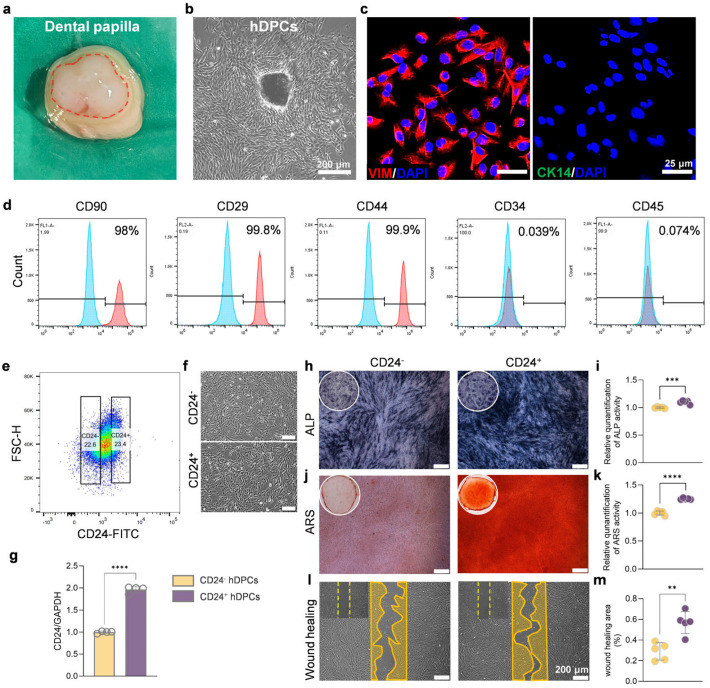
Isolation, characterization, and functional superiority of the CD24^+^ subpopulation. (**a**) Human dental papilla tissue in the third molar is marked with a red dotted line. (**b**) Representative phase-contrast image showed the typical fibroblast-like morphology of primary hDPCs at P3. Scale bar, 200 µm. (**c**) VIM (red) and CK14 (green) immunofluorescence staining of hDPCs. Nuclei were counterstained with DAPI (blue). Scale bar, 25 µm. (**d**) Identification of hDPCs markers CD90, CD29, and CD44, and negligible markers CD34 and CD45 by flow cytometry. (**e**,**f**) Schematic diagram of flow cytometry sorting and representative images of CD24^+^ and CD24^−^ hDPCs. (**g**) RT-qPCR of *CD24* mRNA expression in CD24^+^ and CD24^−^ hDPCs (*n* = 4 independent replicates). (**h**,**i**) Representative images (**h**) and quantification (**i**) of Alkaline Phosphatase (ALP) staining after 7 days of osteogenic induction (*n* = 5 independent replicates). Scale bar, 200 µm. (**j**,**k**) Representative images (**j**) and quantitative analysis (**k**) of Alizarin Red S (ARS) staining after 21 days (*n* = 5 independent replicates). Scale bar, 200 µm. (**l**,**m**) Wound healing assay of CD24^+^ and CD24^−^ hDPCs at 0 and 24 h post-scratch. The circled areas indicate regions of cell migration (*n* = 5 independent replicates). Scale bar, 200 µm. Results are presented as mean ± SD from at least three independent replicates, with statistical significance determined using Student’s *t* test; ** *p* < 0.01, *** *p* < 0.001, **** *p* < 0.0001.

To determine the presence of a CD24^+^ cell subpopulation in hDPCs, dental papilla tissues were isolated from extracted third molars obtained during orthodontic treatment (Figure 1a). Primary hDPCs were extracted via mechanical dissociation combined with type I collagenase digestion. By day 10 of culture, microscopic observation showed cells migrating radially from the tissue explants, exhibiting adherent growth and a typical elongated, spindle-shaped morphology (Figure 1b), consistent with previously reported characteristics of odontogenic mesenchymal stem cells [23,24].

To further characterize cellular identity, immunofluorescence staining for key markers was performed. The results demonstrated expression of the mesenchymal marker VIM, with the absence of the epithelial marker CK14 (Figure 1c), indicating a mesenchymal origin. Furthermore, flow cytometric analysis revealed that the isolated hDPCs highly expressed mesenchymal stem cell surface markers CD90 (98%), CD29 (99.8%), and CD44 (99.9%), while lacking expression of hematopoietic markers CD34 (0.039%) and CD45 (0.074%) (Figure 1d), further confirming their mesenchymal stem cell phenotype.

CD24^+^ and CD24^−^ hDPCs were subsequently isolated by flow cytometric sorting (Figure 1e,f). RT-qPCR analysis confirmed significantly higher *CD24* mRNA expression in CD24^+^ hDPCs compared with the CD24^−^ hDPCs (Figure 1g). Functional assays revealed that CD24^+^ hDPCs exhibited enhanced alkaline phosphatase activity and increased mineralized nodule formation, as demonstrated by ALP and Alizarin Red S staining (Figure 1h–k), indicating superior osteo/odontogenic differentiation potential. In addition, Wound healing assays confirmed significantly enhanced migratory capacity in CD24^+^ hDPCs relative to CD24^−^ hDPCs (Figure 1m).

In conclusion, CD24^+^ hDPCs were successfully isolated and characterized in this study. These cells not only expressed high levels of *CD24* but also exhibited enhanced mineralization capacity and migratory activity, supporting their use as an ideal cellular model for subsequent regenerative studies.

### 3.2. CD24^+^ hDPCs Promote the Proliferation, Migration, and Osteogenic Differentiation of hDPSCs via a Paracrine Pathway

**Figure 2:**
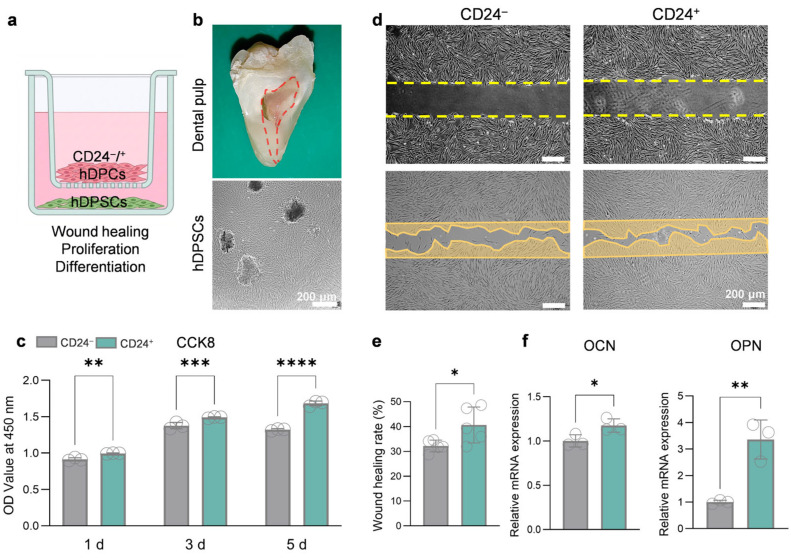
CD24^+^ hDPCs enhance the functions of hDPSCs via paracrine signaling in a co-culture system. (**a**) Schematic diagram of the co-culture transwell system. (**b**) Representative phase-contrast image of isolated primary hDPSCs. Scale bar, 200 µm. (**c**) CCK-8 assay evaluates the pro-proliferative effect of CD24^+^ hDPCs on hDPSCs over 5 days (*n* = 5 independent replicates). (**d**,**e**) Wound-healing assay of hDPSCs co-cultured with CD24^+^/^−^ hDPCs (**d**), with quantified wound healing rates (**e**). The circled areas indicate regions of cell migration (*n* = 5 independent replicates). Scale bar in d, 200 µm. (**f**) RT-qPCR analysis of osteogenic marker genes (OPN, OCN) in hDPSCs after 7 days of osteogenic induction in the co-culture system (*n* = 3 independent replicates). Data are presented as mean ± SD; * *p* < 0.05, ** *p* < 0.01, *** *p* < 0.001, **** *p* < 0.0001.

Dental pulp stem cells are the primary functional cell population responsible for dentin maintenance, repair, and pulp tissue regeneration. Functionally active stem cells persist within the apical region following pulp injury, providing a biological basis for pulp regeneration [25,26,27]. Therefore, to investigate whether CD24^+^ hDPCs regulate the regenerative functions of hDPSCs, hDPSCs were selected as target cells and a Transwell co-culture system was established to assess CD24^+^ hDPCs-mediated paracrine effects on hDPSCs behavior (Figure 2a). Primary hDPSCs were successfully isolated from extracted third molars (Figure 2b). CCK-8 assays showed that co-culture with CD24^+^ hDPCs significantly promoted the proliferation of hDPSCs compared with that of CD24^−^ hDPCs (Figure 2c). The Wound healing further demonstrated that co-culture with CD24^+^ hDPCs enhanced the migratory capacity of hDPSCs (Figure 2d,e). To evaluate effects on osteogenic differentiation, the culture medium in the co-culture system was replaced with osteogenic induction medium. After 7 days of induction, RT-qPCR analysis revealed significant upregulation of osteogenic markers (*OPN* and *OCN*) in hDPSCs co-cultured with CD24^+^ hDPCs (Figure 2f), indicating that CD24^+^ hDPCs enhance the osteogenic differentiation potential of hDPSCs in vitro.

**Figure 3:**
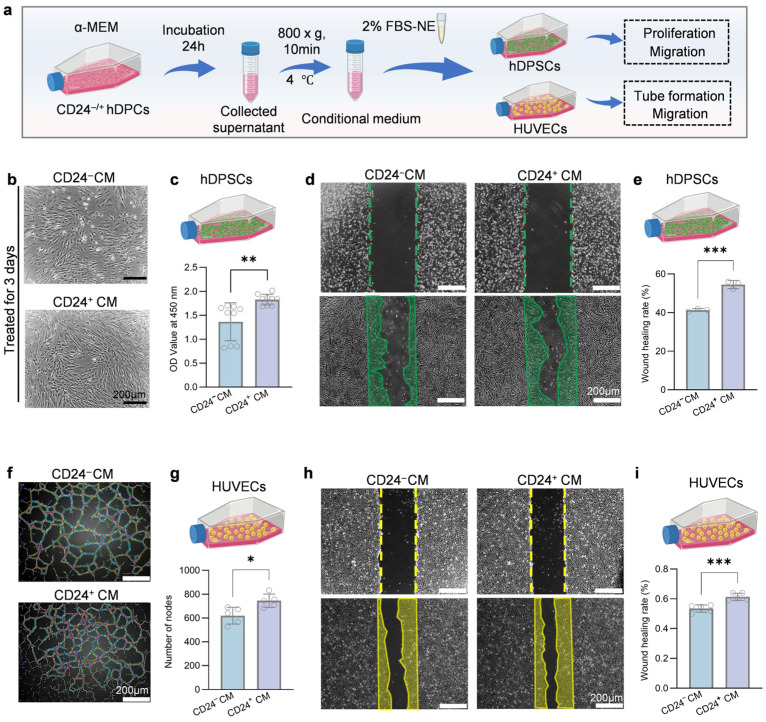
CM from CD24^+^ hDPCs promotes hDPSCs functions and HUVECs angiogenesis. (**a**) Schematic illustration for the preparation of CM from CD24^+^ and CD24^−^ hDPCs. (**b**,**c**) CCK-8 assay (**b**) and representative image (**c**) of hDPSCs cultured with different CMs (*n* = 8 independent replicates). Scale bar, 200 µm. (**d**,**e**) Transwell migration assay (**d**) and quantitative analysis (**e**) of hDPSCs treated with CMs (*n* = 3 independent replicates). Scale bar, 200 µm. (**f**,**g**) Tube-formation assay of HUVECs cultured in different CMs; representative images (**f**) and quantitative analysis of nodes (**g**) (*n* = 5 independent replicates). Scale bar, 200 µm. (**h**,**i**) Wound healing assay of HUVECs treated with different CMs. Representative images (**h**) and quantified healing rates (**i**) (*n* = 5 independent replicates). Scale bar, 200 µm. Results are presented as mean ± SD from at least three independent replicates, with statistical significance determined using Student’s *t* test; * *p* < 0.05, ** *p* < 0.01, *** *p* < 0.001.

To further examine these paracrine effects, CD24^+^ CM and CD24^−^ CM were collected for subsequent experiments (Figure 3a). When hDPSCs were cultured with those CMs, CD24^+^ CM significantly promoted hDPSCs proliferation (Figure 3b,c) and migration (Figure 3d,e) compared with CD24^−^ CM. Given the critical role of angiogenesis in pulp regeneration, the effects of CD24^+^ CM on endothelial cell function were further evaluated. Compared to the control medium, CD24^+^ CM markedly enhanced HUVECs tube formation, as evidenced by significant increases in the number of nodes (Figure 3f,g). In parallel, HUVEC migratory capacity was also significantly increased in the presence of CD24^+^ CM (Figure 3h,i).

Collectively, these results indicate that paracrine factors released by CD24^+^ hDPCs, as modeled by both Transwell co-culture and CD24^+^ CM, significantly enhance hDPSCs proliferation and migration as well as the angiogenic activity of HUVECs. These findings support the notion that CD24^+^ hDPCs help establish a microenvironment conducive to pulp regeneration via paracrine signaling.

### 3.3. CD24^+^ EVs Promote hDPSCs and HUVECs Functions

**Figure 4:**
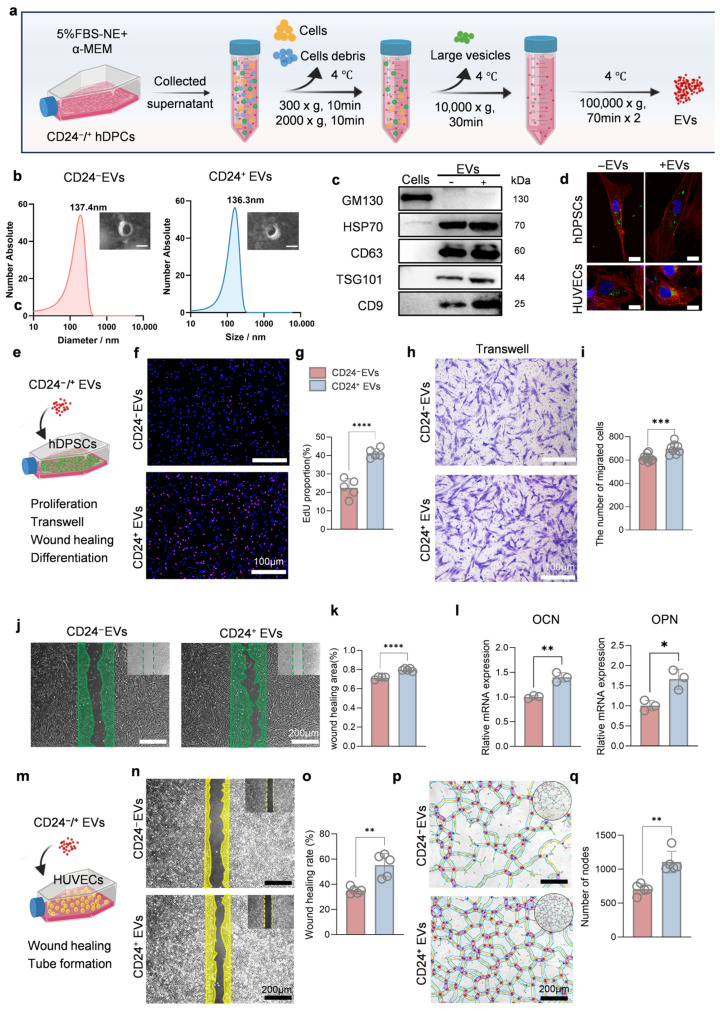
Characterization and in vitro functional validation of CD24^+^ EVs. (**a**) Schematic illustration of the workflow for EVs isolation by ultracentrifugation. (**b**) TEM and NTA of isolated EVs. Scale bar, 100 nm. (**c**) Western blot analysis of exosomal marker proteins (CD63, TSG101, CD9, HSP70) and the negative control marker GM130. (**d**) Cellular uptake assay of DiO-labeled EVs (green) by hDPSCs and HUVECs after co-incubation; the cytoskeleton was stained with phalloidin (red) and nuclei with DAPI (blue). Scale bar, 10 µm. (**e**) Schematic diagram of CD24^+^ EVs regulating hDPSCs function. (**f**,**g**) EdU proliferation assay (**f**) and quantitative analysis (**g**) of hDPSCs treated with CD24^+^ EVs (*n* = 5 independent replicates). Scale bar, 100 nm. (**h**,**i**) Transwell migration assay (**h**) and quantified (**i**) migrated cells confirming the pro-migratory effect of CD24^+^ EVs on hDPSCs (*n* = 5 independent replicates). Scale bars, 100 µm. (**j**,**k**) Wound-healing assay (**j**) and quantitative analysis (**k**) of hDPSCs cultured with different EVs (*n* = 5 independent replicates). Scale bar, 200 µm. (**l**) RT-qPCR analysis of osteogenic gene expression (*OPN*, *OCN*) in hDPSCs treated with CD24^+^ EVs under osteogenic induction (*n* = 3 independent replicates). (**m**) Schematic diagram of CD24^+^ EVs regulating the function of HUVECs. (**n**,**o**) Transwell migration assay (**n**) and quantitative analysis (**o**) of HUVECs treated with CD24^+^ EVs (*n* = 5 independent replicates). (**p**,**q**) Tube formation assay of HUVECs cultured with different EVs; representative images (**p**) and quantitative analysis of nodes (**q**) (*n* = 5 independent replicates). Scale bar, 200 µm. Results are presented as mean ± SD from at least three independent replicates, with statistical significance determined using Student’s *t* test; * *p* < 0.05, ** *p* < 0.01, *** *p* < 0.001, **** *p* < 0.0001.

EVs have been widely recognized as important components of paracrine communication. To evaluate the potential role of CD24^+^ EVs in modulating the functions of hDPSCs and HUVECs, we isolated EVs from the culture supernatants of CD24^+^ and CD24^−^ hDPCs using ultracentrifugation (designated CD24^+^ EVs and CD24^−^ EVs, respectively) (Figure 4a). TEM showed that the isolated vesicles exhibited a typical cup-shaped morphology with diameters below 150 nm and intact membrane structures. NTA further confirmed that the size distributions of both CD24^+^ and CD24^−^ EVs were consistent with typical exosomal profiles (Figure 4b). Moreover, Western blotting demonstrated that both CD24^+^ and CD24^−^ EVs expressed exosomal markers CD63, TSG101, CD9, and HSP70, while the negative marker GM130 was not detected (Figure 4c and Figure S4). Together, these data indicate that high-purity EVs were successfully isolated and are suitable for subsequent functional studies.

To assess cellular uptake of CD24^+^/^−^ EVs, hDPSCs and HUVECs were incubated with DiO-labeled EVs. After 6 h, strong intracellular fluorescence signals were detected in both cell types, confirming efficient internalization of EVs (Figure 4d and Figure S1). Based on hDPSCs proliferation screening, 20 μg/mL was identified as an effective concentration and was therefore used for subsequent in vitro experiments (Figure S2). We next examined the biological effects of CD24^+^ EVs on hDPSCs functions (Figure 4e). EdU staining showed that CD24^+^ EVs significantly promoted the hDPSCs proliferation (Figure 4f,g). Transwell and Wound healing assays further revealed a marked enhancement in hDPSCs migration in the CD24^+^ EVs group (Figure 4h–k). After osteogenic induction, RT-qPCR analysis demonstrated significant upregulation of osteogenesis-related genes in hDPSCs treated with the CD24^+^ EVs (Figure 4l).

We then evaluated the angiogenic effects of CD24^+^ EVs in HUVECs (Figure 4m). CD24^+^ EV treatment significantly enhanced HUVECs migration compared with CD24^−^ EVs (Figure 4n,o). Consistently, in the tube formation assay, CD24^+^ EVs induced a marked increase in the number of nodes, indicating augmented pro-angiogenic activity (Figure 4p,q).

Taken together, these data indicate that EVs derived from CD24^+^ hDPCs recapitulate key functional advantages of their parental cells, promoting hDPSCs proliferation, migration, and osteogenic differentiation as well as HUVECs angiogenic activity, and provide a foundational basis for subsequent in vivo studies on the role of CD24^+^ EVs in supporting the formation of vascularized, functionally relevant pulp-like tissue.

### 3.4. CD24^+^ EVs Drive Well-Structured Pulp Regeneration and Odontoblastic Differentiation

**Figure 5:**
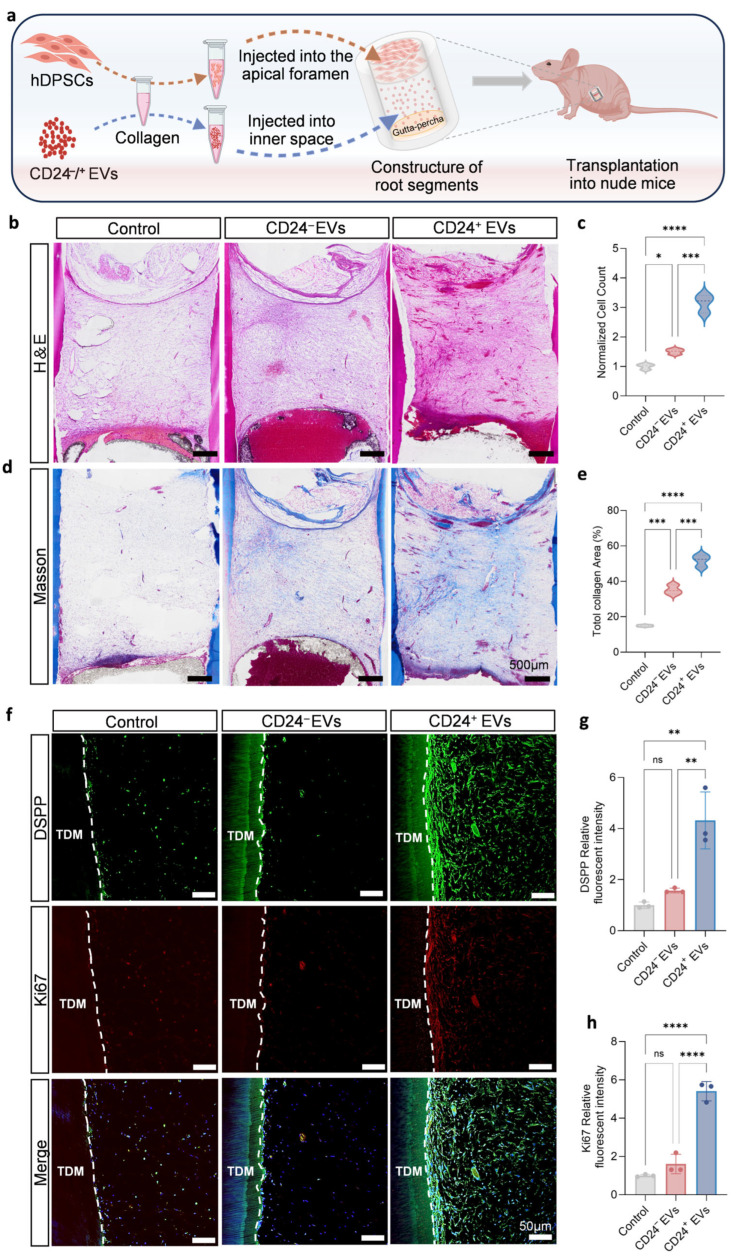
CD24^+^ EVs promote well-structured pulp-like tissue regeneration and odontoblastic differentiation in vivo. (**a**) Schematic illustration of the spatially separated TDM-based ectopic pulp-regeneration model in nude mice, illustrating the compartmentalized loading of EVs and cells. (**b**,**c**) Representative H&E staining (overview) of regenerated tissues at 4 weeks post-implantation (*n* = 5 independent replicates). scale bar, 500 µm. (**d**,**e**) Representative Masson’s trichrome staining (overview) showing regenerated collagen in three groups (*n* = 5 independent replicates). Scale bar, 500 µm. (**f**–**h**) Immunofluorescence staining of regenerated tissues for odontogenic marker DSPP (green) and proliferation marker Ki67 (red); nuclei counterstained with DAPI (blue). The white dotted line marks the boundary between TDM and regenerated tissue (*n* = 3 independent replicates). Scale bar, 50 µm. Results are presented as mean ± SD from at least three independent replicates, with statistical significance determined using one-way ANOVA with Tukey’s post hoc test; * *p* < 0.05, ** *p* < 0.01, *** *p* < 0.001, **** *p* < 0.0001, ns, not significant.

To validate the pulp regeneration capacity of CD24^+^ EVs in vivo, we established a TDM-based subcutaneous ectopic pulp regeneration model in nude mice [21]. In this model, CD24^+^ EVs and hDPSCs were sequentially loaded into a TDM chamber in spatially separated compartments to approximate their physiological distribution (Figure 5a), and we use the collagen group to represent the foundational regenerative capacity. Three experimental groups were established: (1) Control: collagen; (2) CD24^−^ EVs: collagen-loaded CD24^−^ EVs; (3) CD24^+^ EVs: collagen-loaded CD24^+^ EVs. After 4 weeks of subcutaneous implantation, newly formed tissues were observed in all groups. H&E staining showed that the CD24^+^ EVs group contained denser regenerated tissue with significantly higher cell density per unit area, consistent with enhanced cell migration and proliferation (Figure 5b,c). Masson’s trichrome staining revealed robust collagen matrix in the CD24^+^ EVs group, indicating more active extracellular matrix deposition and remodeling (Figure 5d,e). Additionally, immunofluorescence staining demonstrated significantly increased expression of odontoblastic differentiation marker DSPP and proliferation marker Ki67 in the CD24^+^ EVs group (Figure 5f–h), supporting a role for CD24^+^ EVs in promoting odontoblastic differentiation. Collectively, these findings suggest that CD24^+^ EVs not only facilitate cellular migration and the formation of more cellular regenerated tissue but also enhance odontoblastic differentiation, thereby contributing to the generation of well-organized pulp-like tissue in vivo.

### 3.5. CD24^+^ EVs Promote Vascularization in Regenerated Pulp Tissue

**Figure 6:**
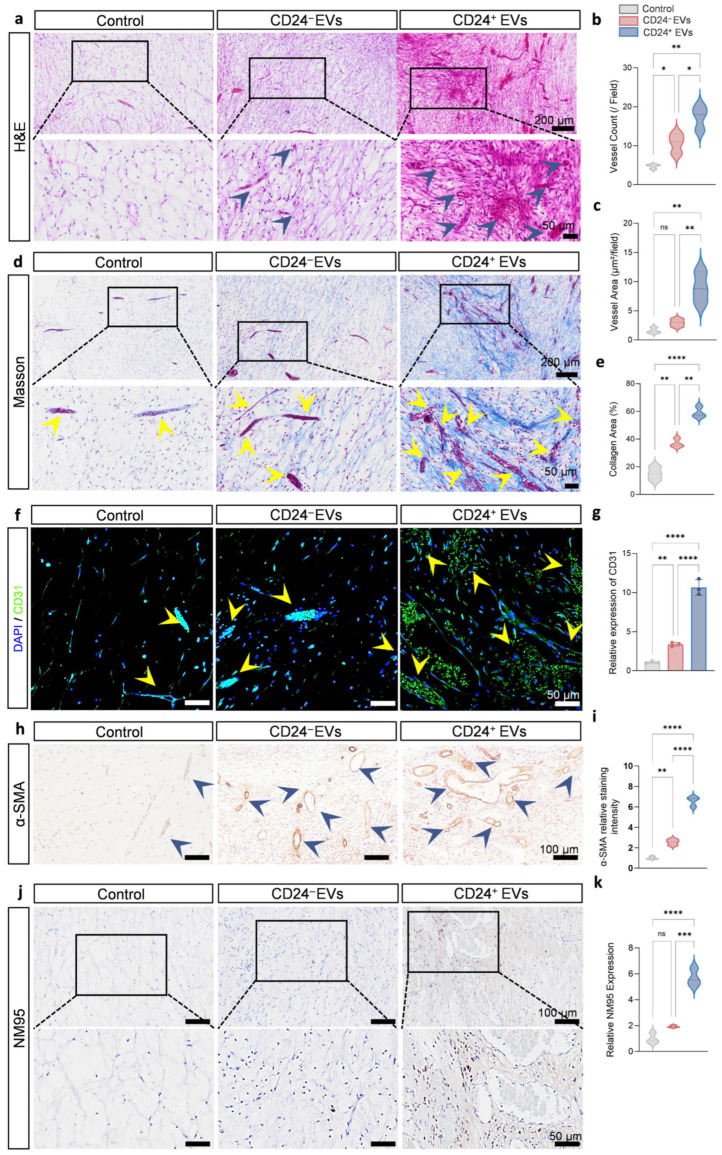
In vivo characterization of vascularization within regenerated pulp-like tissue induced by CD24^+^ EVs. (**a**–**c**) Representative H&E-stained images and quantitative analysis of vascular density and area in regenerated pulp-like tissue. The blue arrows indicate the blood vessels (*n* = 5 independent replicates). (**d**,**e**) Representative Masson staining images showing collagen distribution (blue) and vascular structures (arrows). The yellow arrows indicate the blood vessels (*n* = 5 independent replicates). (**f**,**g**) Immunofluorescence staining (**f**) and quantitative analysis (**g**) of endothelial marker CD31 (green), nuclei counterstained with DAPI (blue). Scale bar, 50 µm. (**h**,**i**) Immunohistochemical staining for α-SMA (brown) to visualize pericyte coverage around vessels. The blue arrows indicate vascular structures (*n* = 3 independent replicates). Scale bar, 100 µm. (**j**,**k**) Immunohistochemical staining using NM95 (brown) to trace the implanted hDPSCs in regenerated tissue (*n* = 3 independent replicates). Results are presented as mean ± SD from at least three independent replicates, with statistical significance determined using one-way ANOVA with Tukey’s post hoc test; * *p* < 0.05, ** *p* < 0.01, *** *p* < 0.001, **** *p* < 0.0001, ns, not significant.

Given the essential role of angiogenesis in pulp tissue survival and regeneration, vascularization within regenerated pulp tissues was further evaluated. H&E staining showed that the CD24^+^ EVs group contained a higher number of blood vessels and a larger vessel area per field, approximately threefold greater than in the CD24^−^ EVs group (Figure 6a–c). Masson’s trichrome staining further revealed more abundant and better-organized collagen fiber deposition in the CD24^+^ EVs group, indicating more active extracellular matrix remodeling (Figure 6d,e). CD31 immunofluorescence analysis demonstrated a significantly higher density of vascular endothelial cells and more organized tubular structures in the CD24^+^ EVs group compared with the CD24^−^ EVs and control groups, again reaching roughly threefold the levels observed in the comparison groups (Figure 6f,g and Figure S3). α-SMA immunohistochemistry showed increased perivascular α-SMA^+^ cell coverage around neovessels in the CD24^+^ EVs group, consistent with a more mature and potentially more stable microvascular network (Figure 6h,i).

This optimized vascularized microenvironment is expected to provide favorable conditions for directional cell migration and colonization. Using human-specific antibody NM95 for lineage tracing, we observed that increased localization of hDPSCs within the vascular-rich central region in the CD24^+^ EV group, suggesting that CD24^+^ EVs enhance migration of apical hDPSCs toward the regenerative core (Figure 6j,k). These observations support a model in which CD24^+^ EVs do not merely promote angiogenesis and cell migration as independent processes, but help construct a vascularized niche that more efficiently recruits and supports regenerative cell populations.

Taken together, these histological and immunohistochemical findings indicate that CD24^+^ EVs coordinately enhance vascularization and cell migration in vivo, supporting their potential as key acellular mediators in strategies aimed at regenerating functional dental pulp.

## 4. Discussion

**Figure 7:**
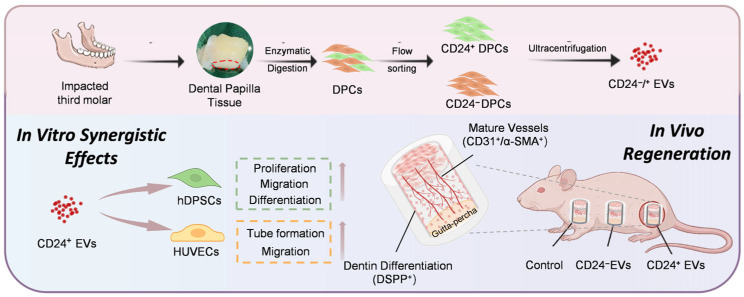
Schematic illustration of CD24+ EVs isolation and in vitro and in vivo regenerative effects.

This study suggests that CD24^+^ EVs can promote pulp-like tissue regeneration in a cell-assisted EV approach. CD24^+^ EVs significantly enhanced hDPSCs proliferation, migration, and osteo/odontogenic differentiation in vitro and concurrently augmented endothelial tube formation and migratory capacity. In a TDM-based ectopic pulp regeneration model, CD24^+^ EVs increased the migration of apically loaded hDPSCs to the regenerative site, supported robust angiogenesis, and led to the formation of densely cellular, highly vascularized pulp-like tissue. In combination, these findings support our central hypothesis that EVs derived from functionally superior cell subpopulations act as signaling mediators in pulp regeneration and highlight CD24^+^ EVs as a promising acellular modality for promoting functional and vascularized pulp-like tissue regeneration (Figure 7).

Our research explores the potential link between cellular heterogeneity and EV heterogeneity. We propose that CD24^+^ EVs inherit the specialized functional attributes of their parent cells, thereby facilitating subsequent regenerative processes. This is supported by the observation that CD24^+^ EVs enhanced both angiogenesis and osteo/odontogenic differentiation, thereby acting on two critical axes of pulp regeneration [28,29]. The nascent vascular network not only supplies nutrients to the regenerating tissue, but endothelial cells within this network can also release trophic factors that cooperate with CD24^+^ EVs-mediated signals to shape a pro-regenerative microenvironment, potentially accelerating the formation of functionally competent pulp-dentin-like tissue.

The spatially separated TDM-based regeneration model [21] used in this study provided a useful platform for assessing the in vivo regenerative potential of EVs. NM95 staining showed increased migration of DPSCs from the simulated apical region into the central pulp chamber in the CD24^+^ EVs group. Compared with traditional strategies that directly place cells into the defect area, this EV-mediated guidance paradigm aims to more closely mimic the natural repair process and may reduce potential risks associated with direct cell transplantation. It is speculated that specific integrins or chemokines on the surface of CD24^+^ EVs may contribute to this targeted stem cell migration [30,31,32], which will be an important focus of future research.

Vascularization is crucial for tissue regeneration, serving not only to nourish the newly formed tissue but also to provide a microenvironmental framework that guides cell behavior and tissue maturation [33]. Current vascularization strategies for tissue regeneration, such as autologous or allogeneic preformed vascular networks, endothelial cell transplantation, or delivery of single growth factors (e.g., VEGF), while effective, are generally plagued by operational complexity, high cost, short half-life of the factors, and susceptibility to loss of activity [34,35]. In this context, our study suggests that CD24^+^ EVs offer a promising cell-assisted EV approach to enhance vascularization. CD24^+^ EVs function as a multifactorial signaling platform capable of initiating and coordinating multiple key steps of angiogenesis: functionally, they significantly enhanced endothelial cell migration and tube formation; structurally, they promoted the formation in vivo of mature CD31^+^ vascular networks covered by α-SMA^+^ pericytes. Notably, the coordinated induction of both angiogenesis and osteo/odontogenic differentiation by CD24^+^ EVs establishes a supportive microenvironment that accelerates tissue maturation and functionalization. Therefore, this study supports the use of CD24^+^ EVs as a promising strategy to drive vascular network formation in the context of pulp-like tissue regeneration.

Despite these encouraging findings, several limitations should be acknowledged. First, we did not include EV-depleted CM controls or pharmacological and genetic inhibition of EV biogenesis or uptake in this study; therefore, we interpret our results as demonstrating EV-associated bioactivity rather than definitive EV-exclusive mediation. Although the multifunctional effects of CD24^+^ EVs were demonstrated, the specific key molecules responsible for these effects have not yet been identified. Future studies should prioritize functional blockade assays and high-throughput proteomic or miRNA profiling to identify key active components, followed by targeted gain- and loss-of-function experiments to validate their roles. Furthermore, while the CD24^+^ EVs group exhibited increased vascular marker-positive structures, these observations do not constitute direct evidence of functional vascular integration or anastomosis with host circulation. The absence of functional perfusion analyses, such as microangiography or Doppler-based assessments, represents an additional limitation of the current study and should be addressed in future work. Moreover, some experiments in the present study were conducted with relatively small sample sizes due to practical experimental constraints; therefore, these results should be interpreted with appropriate caution and warrant confirmation in future studies with larger cohorts. Additionally, the ectopic subcutaneous model used in this study [21], while suitable for assessing regenerative potential, does not fully recapitulate the complex inflammatory microenvironment characteristic of an infected pulp chamber in situ [36,37,38], and the TDM scaffold-only and unsorted hDPCs EVs group were not included in the current experimental design. Therefore, incorporating scaffold-only and unsorted hDPCs-EVs groups, together with comprehensive cost–benefit considerations, will be an important direction for future studies. Finally, subsequent research should include the establishment of inflammatory models, both in vitro and in vivo, to evaluate the ability of CD24^+^ EVs to preserve or restore stem cell function under pathological conditions, together with the development of orthotopic pulp regeneration models in large animals to more rigorously assess translational feasibility.

Taken as a whole, this study identifies CD24^+^ EVs as a promising approach for pulp-like tissue regeneration and, more broadly, advances a functional subpopulation-based paradigm for cell-assisted EV therapy. By selectively harnessing EVs derived from highly potent cell subpopulations, it may be possible to achieve superior regenerative efficacy while reducing risks associated with direct cell transplantation. This work provides a conceptual and experimental basis for the development of safe, efficient, and potentially off-the-shelf biologic agents for pulp regeneration, and supports a shift in EV-based therapeutic strategies from bulk heterogeneous preparations toward functionally defined EV populations.

## 5. Conclusions

In summary, CD24^+^ EVs enhance key biological processes involved in pulp-like tissue regeneration, including cell migration, odontoblast-like differentiation, and the establishment of vascular networks with features of structural maturation. These findings support the concept that EVs originating from functionally defined cellular subpopulations possess distinct advantages for tissue regeneration and provide experimental evidence and a mechanistic rationale for developing cell-assisted EV strategies for pulp regeneration. Taken together, this work demonstrates the feasibility of utilizing subpopulation-derived EVs for dental pulp repair. These findings provide an experimental basis for a potential transition from conventional cell transplantation toward more precise cell-assisted EV interventions. This approach aims to leverage regenerative potential while mitigating the risks associated with whole cell transplantation. More broadly, this study offers a framework for subpopulation-guided regenerative therapies. Although an ectopic model was utilized in this study, the results provide a foundation for developing safe and controllable treatments in regenerative endodontics. Future research will focus on verifying this strategy in large animal orthotopic models to further evaluate its clinical translational potential.

## Data Availability

The original contributions presented in this study are included in the article/Supplementary Materials. Further inquiries can be directed to the corresponding authors.

## Supplementary Materials

The following supporting information can be downloaded at: https://www.mdpi.com/article/10.3390/biom16030390/s1. Additional data supporting this study are available in the Supplementary File. The supplementary materials include the following: Figure S1, Internalization of DiO-labeled CD24^+^ EVs by hDPSCs and HUVECs; Figure S2, Dose-response effect of CD24^+^ EVs on hDPSCs viability; Figure S3, Immunofluorescence staining of CD31 reveals a thicker, more organized vascular network in the CD24^+^ EVs group; Figure S4, Original Western blot images for Figure 4c; and Table S1, Primers used for RT-qPCR amplification in this study.
