# Supplementary material for: Extracellular Vesicles Derived from Human CD24+ Dental Papilla Stem Cells Promote Vascularized Dental Pulp Regeneration
Source: Biomolecules. 2026 Mar 5;16(3):390. doi: 10.3390/biom16030390 (PMC13023677; doi:10.3390/biom16030390)
Supplement: Supplementary file 1 [file biomolecules-16-00390-s001.zip › biomolecules-4163900-supplementary.pdf]

Extracellular Vesicles Derived from Human CD24⁺ Dental Papilla Stem Cells Promote Vascularized Dental Pulp Regeneration

Jie Li, Tian Chen, Cheng Liang, Peini Lin, Weidong Tian, Zhi Liu* and Lei Liu*

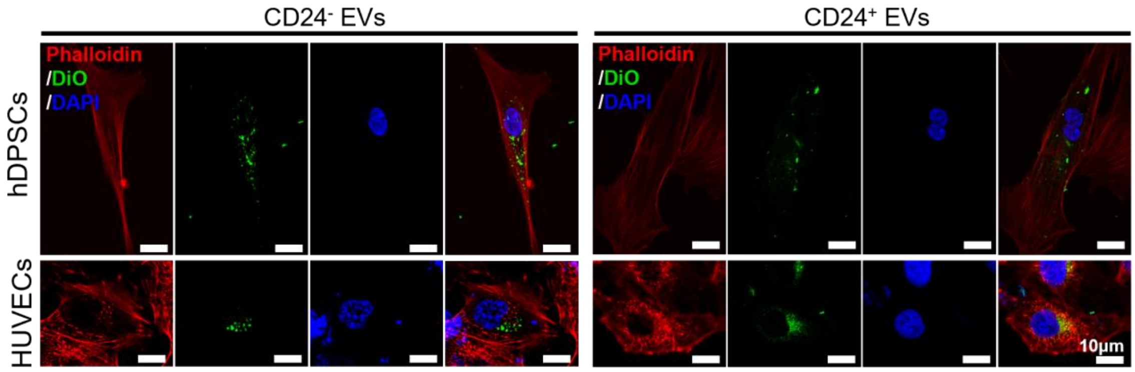

Figure S1. Internalization of DiO-labeled $CD24^+$ EVs by hDPSCs and HUVECs.

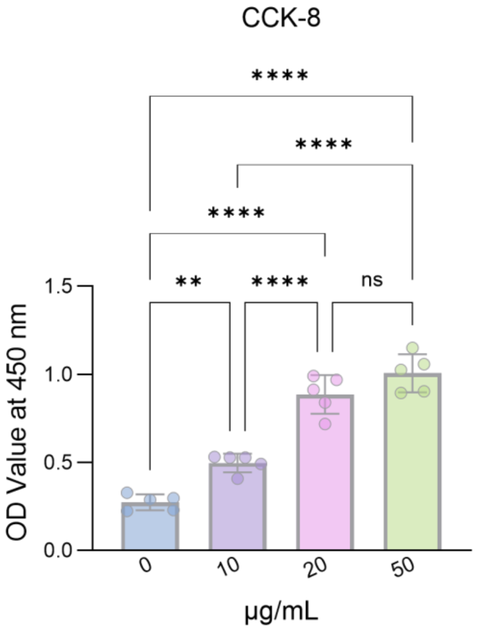

Figure S2. Dose-response effect of $CD24^+$ EVs on hDPSCs viability.

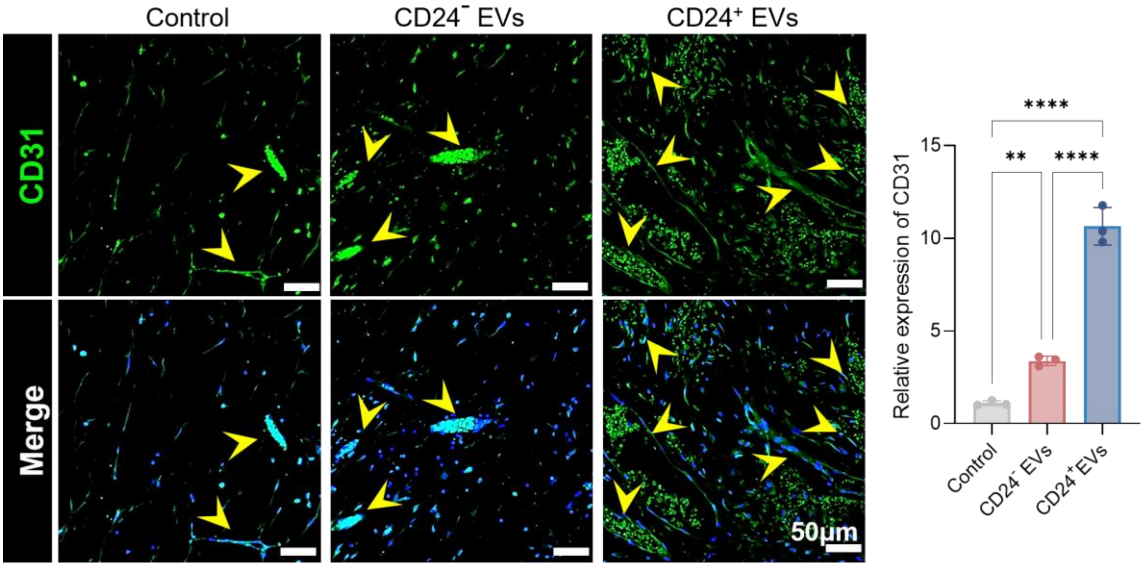

Figure S3. Immunofluorescence staining of CD31 reveals a thicker, more organized vascular network in the CD24⁺ EVs group.

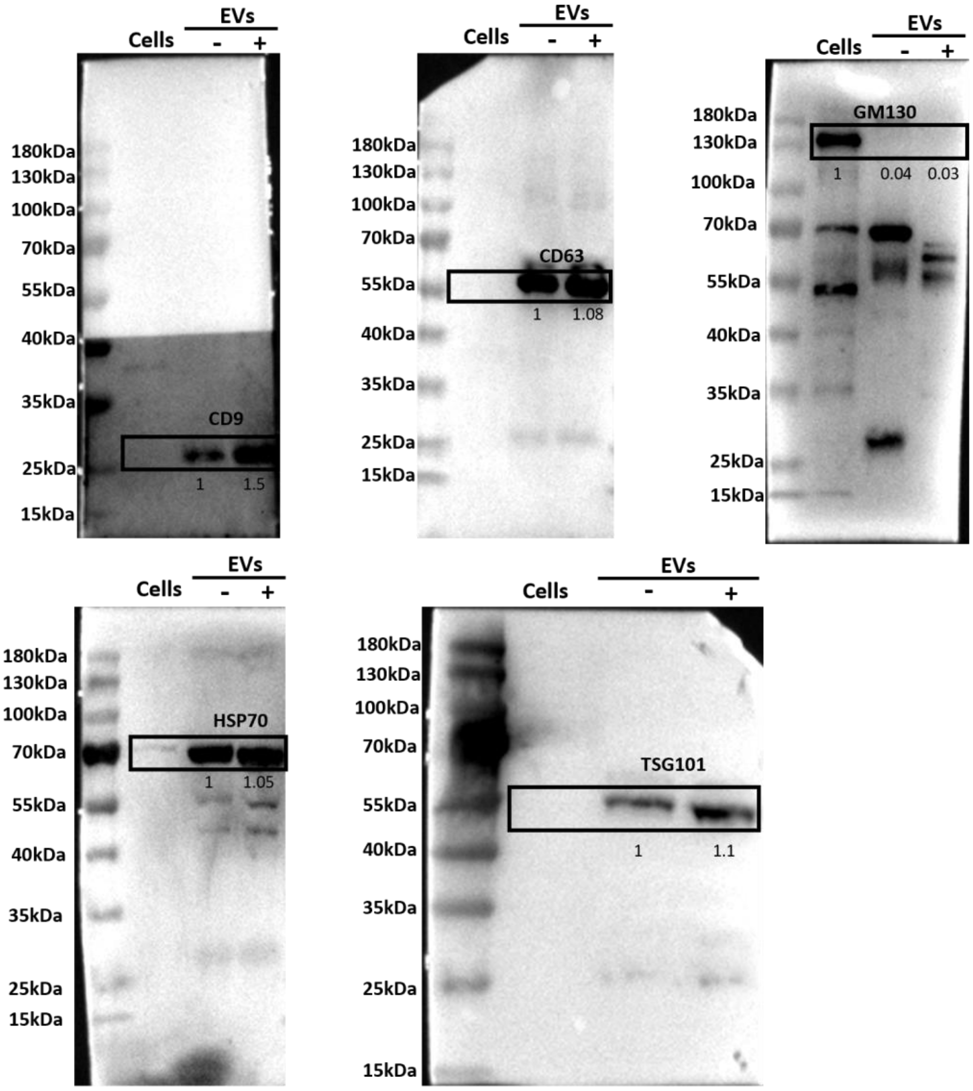

Figure S4. Original Western blot images for Figure 4c.

Table S1: Primers for the amplification in this study.

primers	Sequence（5'— 3'）
CD24-F	CTCCATTCCACAATCCCATC
CD24-R	GAAGGAGAGGCAACATCCAA
OPN-F	CAGTTGTCCCCACAGTAGACAC
OPN-R	GTGATGTCCTCGTCTGTAGCATC
OCN-F	CTCACACTCCTCGCCCTATT
OCN-R	CCTCCTGCTTGGACACAAA
GAPDH-F	CTTTGGTATCGTGGAAGGACTC
GAPDH-R	GTAGAGGCAGGGATGATGTTCT
